# A role for plant science in underpinning the objective of global nutritional security?

**DOI:** 10.1093/aob/mcy118

**Published:** 2018-07-06

**Authors:** Cathie Martin

**Affiliations:** Department of Metabolic Biology, John Innes Centre, Norwich Research Park, Norwich, UK

**Keywords:** Nutritional security, nutritional insufficiency, plant-based foods, phytonutrient, microbiota, inflammation, obesity, chronic disease

## Abstract

**Background:**

The challenges of achieving global food security have become more demanding as scientists have realized that not only calorie content but also food composition and colonic microbial content impact our health and well-being, dramatically. The ways that the nutrients we consume affect our health are highly complex due to the diversity of what we eat, the varying digestibility of what we eat, the changing composition and functioning of each individual’s gut microbiota, the differences in absorption and bioavailability of the nutrients we eat, the differences in responses between individuals to what they eat and the multi-fold mechanisms of action that nutrients have on our health.

**Perspective and Conclusions:**

It has been accepted for more than 50 years that diets rich in plants, particularly fruit and vegetables, protect health, and yet such diets have declined, with lower fruit and vegetable content and much more cheap, sugary, oily, processed foods, over the same period. These dietary shifts have had a marked impact on the incidence of chronic diseases: obesity, metabolic diseases, type 2 diabetes and cardiovascular diseases. Greater support for research into the ways that plant-based foods impact health will be essential for changing dietary patterns to protect health and to achieve global nutritional security.

## INTRODUCTION

When I was young one of the major issues that drove life in my family was responding to international appeals for charitable assistance. My mother worked tirelessly for charities, collecting blankets, clothes and food to be shipped to emergency areas, and at Christmas and Easter collected money to support people in places of famine. The aspiration for the money collected was always to provide water and food so that people had ‘enough to eat’. Her efforts and those of many others were founded on two early principles in famine relief which emerged from the Indian Famine Codes of 1880 and greatly influenced European philosophy on how to deal with famine. The first was that relief should not be so generous as to encourage dependency among recipients and the second was to provide relief without weakening the donor’s economy by intervening or participating in food-grain markets (Hall-Matthews, 1998). Consequently, 50 years ago, our concept of famine relief was based on immediate provision of calories for communities, particularly those afflicted by drought or war, where crops had failed. In 1974 the World Food Conference defined food security solely in terms of yield.

The scientific world embraced the need for increasing agricultural productivity to meet the needs of a burgeoning world population also facing substantial changes in climate. To offer long-term solutions to famine, higher yielding crops were developed during the ‘Green Revolution’ for developing countries and facilitated the alleviation of hunger, but brought with them an increasing dependency of many on a few, calorie-rich but nutritionally poor, staple crops. This dependency on staples gave rise to ‘hidden hunger’ caused by deficiencies in micronutrients (particularly iron and zinc) and essential vitamins (especially provitamin A) in diets based heavily on foods such as white corn, cassava or rice (von Grebmer *et al.*, 2014). With these developments came changes in our understanding of the relationships between nutrition and hunger, and in 1996 the World Food Summit redefined food security as existing ‘when all people, at all times, have physical and economic access to sufficient, safe and nutritious food to meet their dietary needs and food preferences for an active and healthy life’.

Meanwhile, measures taken to address ‘the food problem’ in the USA in the early 1970s, when food price inflation threatened to make the cost and availability of food top issues on the national agenda, gave rise to new nutritional challenges in the developed world. Policy changes under Richard Nixon shifted subsidies from farmers to investments in programmes that boosted yields from a few commodity crops (principally corn and soy in the USA). This allowed agro-industrialization and a dramatic fall in crop prices. Farmers had to produce more in order to break even. The price of many foods fell, especially processed foods and sweetened beverages that could be made from corn or soy. At the same time the price of fresh foods such as fruit and vegetables increased substantially, in relative terms. In breeding crops for increased yield, many important traits including flavour and nutritional value were lost. This focus on commodity crops meant that many of the nutritional advantages of diverse, fruit and vegetable-rich diets were lost. In addition, the processing of commodity crops into vegetable oils, which were frequently hydrogenated to form trans-fats in margarines and baked goods or for products for sweetening such as high fructose corn syrups (HFCS), meant that the consumption of low-cost, health-challenging, anti-nutritionals increased in line with the increased consumption of processed foods.

As a consequence of these changes, problems of nutritional insufficiencies are not restricted to the developing world. Currently, just 17 plant species are consumed as 90 % of the global human diet, meaning that many diets, in both developing and developed countries, lack adequate vitamins, micro-nutrients and health-promoting phytonutrients. The strength and pervasiveness of fast-food industries in developed countries, the relative weakness of their horticultural sectors, the increasing cost of fruit and vegetables relative to convenience foods and the addictive nature of soft, palatable diets (Gearhardt *et al*., 2009, 2011) have all contributed to a general decline in the nutritional value of diets worldwide, and a paucity of fruit and vegetables in modern, Western diets. Following the financial crisis, there has been rising food insecurity in European and North American countries which has brought with it additional nutritional insecurity (Seligman and Schillinger, 2010; Loopstra *et al*., 2015, 2016) People without enough money to buy food reduce the variety of foods in their diet and concentrate on a few low-cost, energy-dense, but nutritionally poor foods including those with added refined carbohydrates, sugars and fats which are cheaper than nutritionally rich fruits, vegetables and dairy products. These dietary shifts have had profound effects on the incidence and management of obesity, hypertension, diabetes, and other diet-sensitive chronic diseases (Seligman and Schillinger, 2010). Nutritional insecurity is now a global problem, and underpins much of the increase in chronic disease, world-wide. Yet measures to address these issues are based, almost exclusively, on expensive public information campaigns that recognize the importance of fruit and vegetables in the diet, but which ultimately have little impact on dietary patterns or behaviours (Rekhy and McConchie, 2014). Towards this end, nutrition security has been linked to food security in the UN’s Sustainable Development Goals – Goal 2: end hunger, achieve food security and improved nutrition, and promote sustainable agriculture (Griggs *et al.* 2013).

Increases in the incidence of chronic disease have accompanied dietary shifts towards nutrient-poor, refined sugar and fat-replete, Western diets, including increases in cardiovascular diseases (CVDs), metabolic diseases, type 2 diabetes and certain cancers, particularly steroid-hormone-related cancers, linked to rising levels of obesity. Yet, many remain reluctant to acknowledge the contribution of diet to protection from chronic disease (https://well.blogs.nytimes.com/2015/08/09/coca-cola-funds-scientists-who-shift-blame-for-obesity-away-from-bad-diets;Nestle, 2013), and this philosophical bias has been summarized by Wendell Berry: ‘People are fed by the food industry that pays no attention to health and treated by the medical industry that pays no attention to food’ (Berry 1993). The failure of diverse agencies to recognize the importance of diet in protecting health is reflected in a general lack of support for investigating the role of plant-based foods in the diet.

## WHAT ROLES DO PLANTS PLAY IN OUR DIET?

Plants provide us with macro-nutrients (carbohydrates, lipids and proteins) in our diet. Although many consumers in developed countries obtain most of their protein from animal products, there are large population groups that are dependent on plant sources of protein. Protein obtained from pulse crops is significantly less expensive and its production is markedly more sustainable than animal protein, requiring substantially lower inputs, especially of water (Singh, 2017). Pulse crops are of particular importance in India because of the high proportion of vegetarians, variously estimated as between 20 and 42 % of the population. There are as many as 500 million people in India solely dependent on non-animal protein sources. Efforts are now being made to increase the availability of plant proteins in the developed world, because plant protein sources (such as legumes) have the lowest environmental production costs, while at the same time demonstrate the highest density of nutrients. Support for such efforts is demonstrated by the funding of consortia under the Horizon 2020 programme such as the TRUE project - transition paths to sustainable legume-based systems in Europe (https://www.true-project.eu/).

Plants provide most essential micronutrients (vitamins A, B and C, some vitamin D, E and K) and most of our essential mineral micronutrients (Martin *et al.*, 2013). In addition, they provide phytonutrients, which are compounds in plant-based foods that play beneficial roles in the prevention and treatment of disease. These have been recognized as important dietary components contributing to significant protection against chronic diseases only relatively recently. Because such phytonutrients are usually not considered ‘essential’ in the same way as vitamins and micronutrients, they are often missing from nutritional evaluations, and clear dietary recommendations for daily allowances are often not available. Phytonutrients include polyphenols (flavonoids and stilbenoids), carotenoids, plant sterols, polyunsaturated fatty acids (PUFAs) and plant fibres.

## CAN PLANTS OFFER A SOLUTION TO NUTRITIONAL INSECURITY?

The past 30 years have seen development of an enormous body of evidence on the importance of plant-based foods in preventing or reducing the risk of chronic disease. Indices of healthy eating that incorporate high consumption of fruit and vegetables, such as the alternative healthy eating index (McCullough *et al.*, 2002), are positively linked to significant reductions in the risk of major chronic diseases. Many foods are now labelled with nutritional information detailing their content of protein, carbohydrates (sugars), fats and additives, but many health-promoting factors in plant-based foods are still not measured or listed. To secure effective shifts in diets towards consumption of more plant-based foods, actions need to be supported at many levels: by governments, policy-makers, food industries, medical industries and healthcare professionals, breeders, farmers, horticultural industries and food purveyors, such as supermarkets and, most importantly, by funders of scientific research.

To gain support from all sectors necessary to implement these profound changes in dietary patterns, much greater understanding of how plant-based foods and phytonutrients impact our health is essential. This, in itself, is a major challenge because food and the diet–health equation are very complex. Understanding how food works to prevent and protect against disease is challenging. However, integrating new ideas from analytical approaches with understanding why traditional diets are usually healthier than modern ‘Western diets’ should inform our progression towards a better understanding of the complex relationships between diet and health and how best for different societies to achieve nutritional security. We also need a far better understanding of the impact of nutritional insufficiency, rather than nutritional deficiency of foods. Insufficiencies underpin the evidence from epidemiological, intervention, clinical and preclinical studies for the importance of dietary phytonutrients in protecting against chronic diseases such as CVD, obesity, metabolic disease, inflammatory diseases and certain cancers, because phytonutrients are not classified as essential in the diet. Understanding the effects of nutritional insufficiencies will be highly demanding scientifically, because effects are likely to be small and therefore difficult to measure, and mechanisms are likely to be multifold and possibly synergistic with other components of food.

What are the processes in food consumption and digestion that make understanding the role of plants in our diet in health and disease, so complex?

## THE DIVERSITY OF WHAT WE EAT

It might appear very hard to make dietary recommendations that are equally suitable for those living in large cities in the USA as well as those living in urban societies in Africa. However, the ‘Westernization’ of diets and the pervasiveness of fast, convenience foods make the diets of such diverged societies much more similar now than they were 30 years ago. Oily fried food and high-sugar drinks are likely to be major constituents of both, and yet these are foods that are of low nutritional quality, devoid of many phytonutrients, and fuel chronic disease.

Initiatives have been taken in the USA to promote the acquisition of fresh fruit and vegetables from farmers markets and urban agriculture/horticulture initiatives such as the San Francisco Urban Agriculture Alliance (http://www.sfuaa.org/). The diversity of products grown through such initiatives is important for their commercial success, but also key to diets being able to supply all of the health-protecting compounds that plants can provide in the diet. Similar initiatives are being championed in developing countries stimulated by the promotion of cultivation and consumption of a diverse selection of indigenous fruit and vegetables. A good example is the promotion of traditional greens in Kenya which have gained significant popularity over the past 5 years and are more nutritious than introduced greens such as kale, being richer in vitamins (A, C, E, folate), calcium and soluble fibre (Cernansky, 2015). In an ideal situation we would be able to compare the relative benefits of consuming local greens such as African nightshade (*Solanum scabrum*) to introduced greens such as kale (*Brassica oleracea*) using local, nutritional information. Currently, there is access to nutrient composition data, for example through the USDA Food Composition Databases (https://ndb.nal.usda.gov/ndb/) or McCance and Widdowson’s The Composition of Foods integrated dataset and those for other European countries, accessible through the EUROFir Hub (http://www.eurofir.org/food-information/food-composition-databases-2). However, information is often limited to major global commodity products and does not include local or indigenous crop varieties. Access to such databases is also often subject to a subscription, making use by the interested consumer practically impossible.

There have been claims that organically produced food is ‘more nutritious’ and therefore organic cultivation should be encouraged to ensure nutritional security (Reganold and Wachter, 2016). There are very few data to support such claims. A meta-analysis of crops grown organically compared to conventionally (non-organic production) suggested that factors including genotype, year, place, environmental conditions and time of harvest were more important in determining nutritional value than organic cultivation methods (assessed by Fe and Zn micronutrient and carotenoid contents) (Johansson *et al.*, 2014). Even in reports where phenolic contents were significantly enhanced by organic cultivation (probably associated with greater pathogenesis and often with reduced yields) the degree of enhancement was not more than 50 %, meaning that the nutritional benefit of these phytonutrients with reported low bioavailability was likely to be very small (Wang *et al.*, 2008; Wojdyło *et al*., 2013). Organic food is, of course, free from synthetic pesticide residues which, in a sense, may make it more ‘nutritious’ but the consumer should not forget that many anti-nutritionals, such as copper, are still used by organic farmers to protect their crops. As a result of the widespread use of Bordeaux mixture in protecting organic vineyards, the French have detected copper levels of over 1000 ppm (or 0.1 %) in agricultural soils, which is ten times the maximum permitted level in US farming (Kovačič *et al.*, 2013).

## THE VARYING DIGESTIBILITY OF WHAT WE EAT

We consume macronutrients (proteins, carbohydrates and lipids) from many different sources, and different plant-based foods may release their macronutrients at very different rates. Reduced digestibility of foods with high levels of fibre was the basis of the F-plan diet of the 1980s popularized in *The F-Plan Diet* by Audrey Eyton (1983).

These ideas were based on the observations of Denis Burkitt that many Western diseases that are rare in Africa, such as colorectal cancer, are strongly impacted by diet and lifestyle (Burkitt, 1979). Burkitt suggested that fibre was an important dietary component for gut health, a view now widely accepted by nutritionists. The F-plan diet suggested that high fibre reduced the digestibility of the food eaten and prevented the absorption of many macronutrients, especially sugars and fats, so facilitating weight loss while maintaining satiation after high-fibre meals.

Currently the roles of fibre in the diet (Table 1) are believed to be more far-reaching than simply reducing the digestibility of macronutrients. The evidence for health beneficial effects of insoluble plant fibre from epidemiological studies is relatively weak (Martin *et al.*, 2013), but many foods rich in insoluble fibre such as whole grains, corn bran, nuts and seeds, potatoes and the skins from fruit such as apples, bananas and avocados, many green vegetables such as green beans, courgette, celery and cauliflower and some fruits such as tomatoes and kiwi are promoted as part of a healthy diet. Insoluble fibre does not dissolve, nor is it fermented by colonic bacteria. It retains water and so promotes larger, bulkier stools and more regular bowel activity. Insoluble fibre is the main component of fibre that reduces the digestibility of other macronutrients in the food matrix.

**Table 1.** Major dietary sources of fibre

Type of fibre	Dietary sources	Pictures	Properties
Insoluble fibre	Whole grains, corn bran, nuts, seed, skin of apples, bananas, avocados, green vegetables	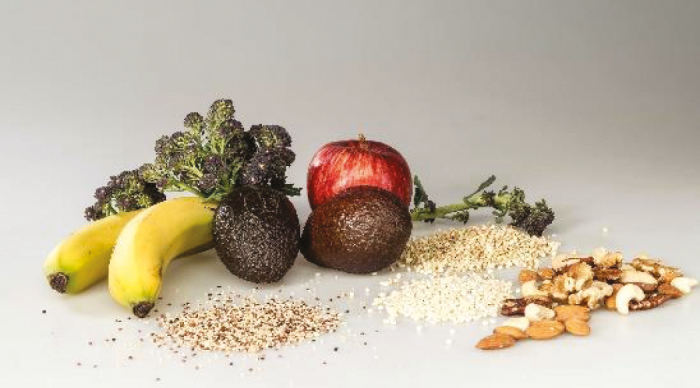	Reduces/slows digestion and improves bowel health
Soluble fibre	Aubergine, okra, peas, beans, oats, rye, barley, berries, apples, bananas, pears, broccoli, root vegetables	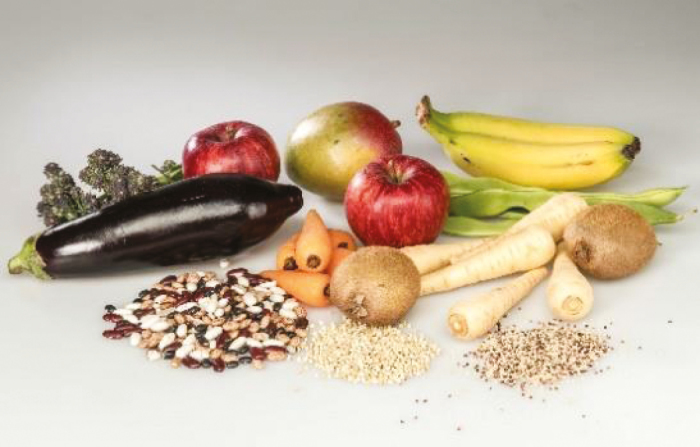	Fermented by microbiota. Lower glycaemic index, beneficial for CVD, obesity and type 2 diabetes
Soluble fibre with probiotic activity	Jerusalem artichoke, leeks, garlic, onions, chicory	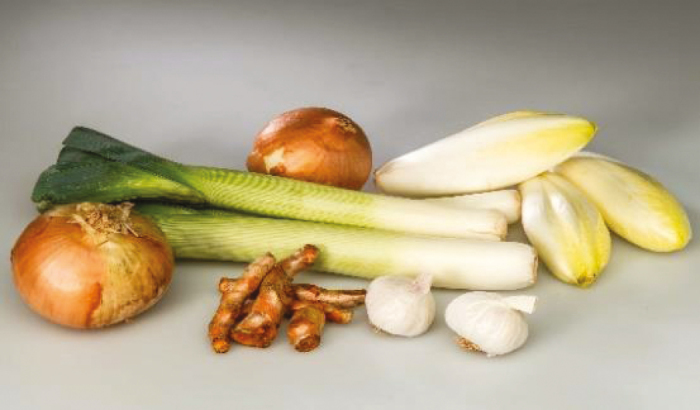	Improves microbiota composition, reduces pH of colon, releases SCFAs, signals satiety
Digestible starch	Cooked white rice, white bread, cooked potatoes	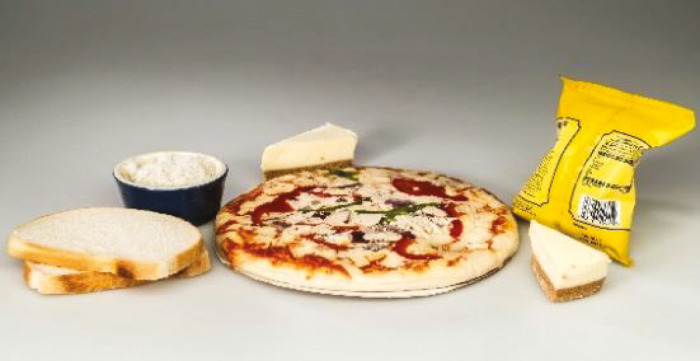	Rapidly digested starch, high glycaemic index, promotes obesity, CVD, type 2 diabetes
Resistant starch	Beans, legumes, whole grains, pasta made from durum wheat, green unripe bananas	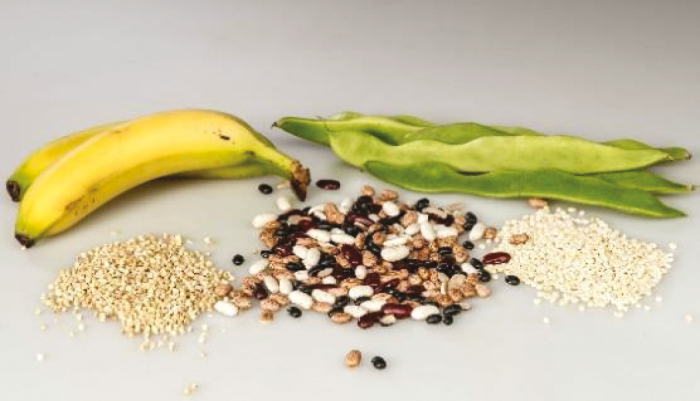	Slowly digested, lower glycaemic index, good for gut health, CVD, and obesity

CVD, cardiovascular disease; SCFA, short-chain fatty acid.

There is considerable evidence from epidemiological studies to support the role of soluble fibre in healthy diets. The inclusion of viscous, soluble fibre in the diet lowers the glycaemic index of foods and has a beneficial impact on type 2 diabetes, risk factors for CVD and obesity (Meyer *et al.*, 2000; Larsson *et al.*, 2009). Soluble plant fibre can be fermented by the bacteria of the colon and health benefits occur as a result. Soluble fibre is present in legumes such as peas and beans, oats, rye and barley, berries, plums, apples, bananas and pears, broccoli and carrots and other root vegetables. Particularly rich sources are aubergine (egg plant) and okra (Kendall *et al.*, 2010; Wanders *et al.*, 2011). The consumption of porridge at breakfast for slow release of energy may depend on the high soluble fibre content of oats.

A subgroup of soluble fibres includes inulin (fructan) fibres and plant-derived oligosaccharides that can act as prebiotics. These types of soluble fibre are not absorbed by the body but are metabolized by colonic microbiota, and promote the growth of Lactobacilli and Bifidobacteria in the colon, which are associated with improved gut health. Some oligosaccharides are associated with the increased production of short-chain fatty acids (SCFAs), which supress inflammation, are preferred energy sources for colonic epithelial cells and signal satiation, which limits food consumption (Slavin, 2013).

Resistant starch has properties similar to those of soluble fibre. Starch consists of two polymers, branched amylopectin and essentially unbranched amylose. Due to the ways that these polysaccharides are arranged in starch granules, branched amylopectin is more easily digested and the linear chains of amylose are broken down more slowly in the gastrointestinal (GI) tract, and it therefore comprises a major fraction of slowly digested starch and resistant starch, type II (Sajilata *et al.*, 2006). Amylose-rich starch is called resistant starch and although it is metabolized to some extent, it has half the caloric value of rapidly digested starch. Resistant starch also induces beneficial changes in the composition of the colonic microbiota and induces production of SCFAs which modulate immune function and lower pH in the gut. The average Western diet includes 3–6 g of resistant starch each day, but traditional diets, such as those studied by Burkitt in East Africa, have considerably higher contents. Good sources of resistant starch are starchy fruits such as green banana and mango. In Uganda, men would traditionally eat matoke dishes of baked green bananas before a long day in the fields, because its high content of resistant starch gave a slow release of energy. Resistant starch and soluble dietary fibre are being promoted for their prebiotic functionalities because of their beneficial effects on gut health and chronic disease (Fuentes-Zaragoza *et al.*, 2011), but also because they are associated with lower glycaemic index foods, suitable for type 2 diabetics.

Other phytonutrients have different bioactivities in different individuals because of differences in the composition of the microbiota in different individuals. Legumes are the source of an important group of bioactive compounds – the isoflavonoids – often referred to as phyto-oestrogens because they bind to oestrogen receptors in animals, and have been assigned many health benefits in humans. They are believed to be important in reducing occurrences of prostate, breast and colon cancers, reducing post-menopausal symptoms and reducing coronary heart disease by reducing low-density lipoprotein (LDL) and increasing high-density lipoprotein (HDL) in plasma (Sacks *et al.*, 2006). Western diets generally have 100-fold lower levels of isoflavonoids compared with Asian diets (due to lower consumption of soy protein products), and epidemiological studies have shown this difference in isoflavonoid intake to be inversely correlated with breast cancer incidence in these two groups (five-fold lower in women on Asian-style diets). Preclinical studies have confirmed the beneficial effects of isoflavonoids in preventing CVD, breast and prostate cancers, and post-menopausal ailments (Dixon and Ferreira, 2002; Cornwall *et al.*, 2004).

The main source of isoflavones in the human diet is soybean, and isoflavones come in several different forms, mainly daidzin and genistin, and small amounts of their aglycones. It is believed that these isoflavones do not have significant bioactivity in their own right, but are metabolized to their aglycones and then to another compound, equol, in the gut by anaerobic colonic bacteria (Setchell *et al.*, 2002). Oestrogen receptors have a very high affinity for equol, greater than that for the plant isoflavones. Thus, the effects of dietary isoflavones in protecting against chronic diseases, particularly steroid-hormone-related cancers, has been attributed to the activity of equol as an oestrogen analogue. However, the ability to produce the more potent isoflavonoid, equol, varies between individuals, which may account for some reported negative responses to isoflavones. This has led to the concept in humans of ‘equol producers’ and ‘non-equol producers’ depending on an individual’s gut microflora (Sacks *et al.*, 2006). Such differences in human subjects must be taken into account in analysing epidemiological and intervention study data, for any results from such studies of dietary isoflavones to be meaningful and for dietary recommendations to be robust. In fact, results from human trials of dietary isoflavones have often been contradictory (Zhan and Ho, 2005), perhaps as a result of failure to score whether participants were equol producers, or not.

These differences between foods in their digestibility and the differences between individuals in their abilities to digest foods make establishing generic relationships between diet and health extremely difficult, and consequently difficult to persuade policy-makers, politicians, and most importantly the food industry and agricultural sectors of the importance of plant-based foods to our diet and health (Nestle, 2013). However, just because these relationships are difficult to prove, does not mean that they do not exist.

## THE ROLE OF THE MICROBIOTA

**Fig. 1.**
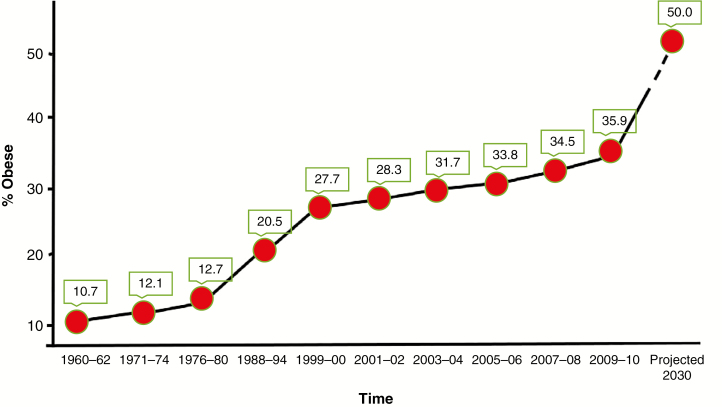
Changing prevalence of obesity (body mass index ≥30) among US adults aged between 20 and 74 years. Data from NHANES (https://www.cdc.gov/nchs/data/hestat/obesity_adult_09_10/obesity_adult_09_10.html#table1).

Many chronic diseases are related directly to obesity, and the global obesity epidemic, which started in the late 1970s, underpins much of the projected increase in mortality from chronic diseases and predictions about the increased prevalence of chronic disease in the near future (World Health Organization, 1998; James, 2009). In the European Union, approx. 60 % of adults and over 20 % of school-age children, equating to around 260 million adults and over 12 million children, are either obese or overweight. In the USA current estimates suggest that more than 35 % of the population is obese with projected associated costs of obesity in terms of healthcare and lost disability-adjusted life-years (DALYs) set to rise to between $48 and $66 billion per annum by 2030 (Wang *et al.*, 2011; Fig. 1). Contrary to many preconceptions, the nutritional quality of food eaten, as opposed to purely its calorific content, predicates significantly on the occurrence of obesity. Consequently, predictions about the future incidence of mortality from chronic diseases are based on the risks to which current adolescents are exposed, primary among which are the poor ‘Western’ diets that have fuelled the obesity epidemic.

The intestinal microbiota has recently been identified as a major modifiable contributor to metabolic disease risk both in laboratory animals and in humans. Distinct profiles of gut bacteria characterize different animal models of obesity and its complications [non-alcoholic fatty liver disease (NAFLD) and type 2 diabetes] as well as in humans who suffer from obesity, type 2 diabetes or NAFLD. The intestinal microbiota of lean and obese individuals differs in composition; obesity is associated with fewer Bacteroidetes and correspondingly more Firmicutes than in lean individuals (Ley *et al.*, 2006). Colonization of germ-free mice with distal gut microbial communities from obese animals results in dramatic increases in body fat despite associated decreases in food consumption (Bäckhed *et al.*, 2004; Turnbaugh *et al.*, 2006). The microbiota of obese individuals are more efficient at extracting energy from a given diet than those of lean individuals. The factors determining the composition of the microbiota are not well established, but any research aimed at understanding the relationship between dietary constituents and obesity, and the mechanisms underpinning the effects of dietary constituents on weight gain, needs to consider and assess the impacts of dietary bioactives on the composition of the gut microbiota.

**Fig. 2.**
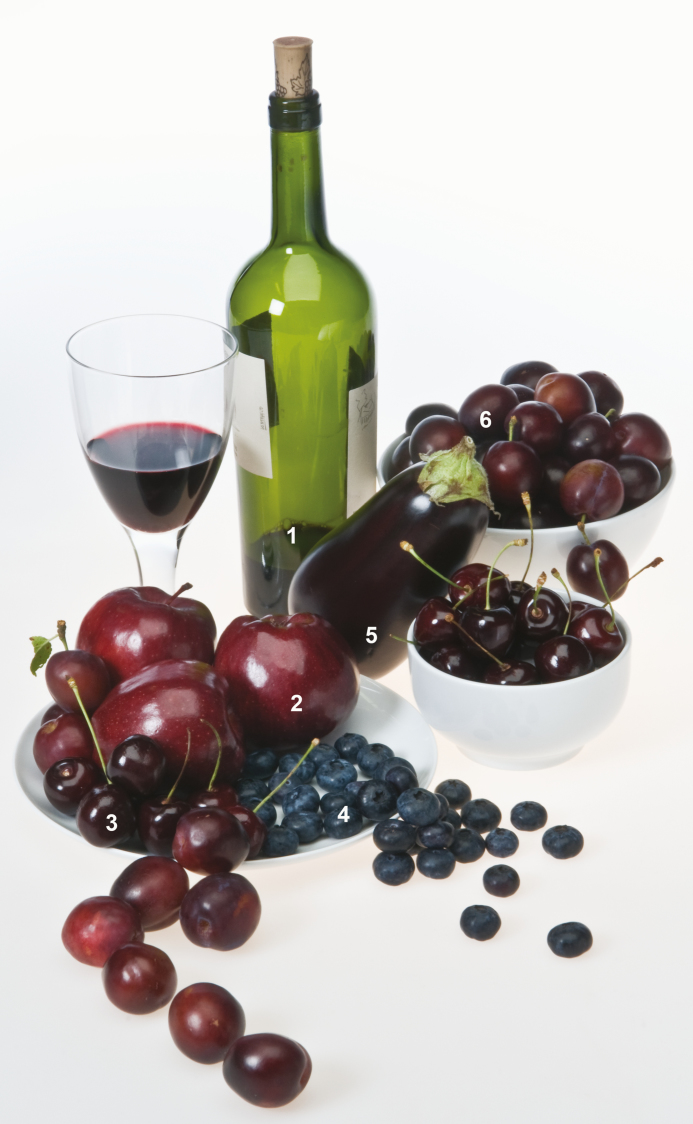
Selection of fruits and fruit products that are rich in anthocyanins. (1) Red wine; (2) red apples; (3) cherries; (4) blueberries; (5) aubergine (egg plant); (6) damson plums.

Many phytonutrients impact the composition of the microbiota. For example, the plant pigments anthocyanins have reported effects on reducing weight gain and obesity in preclinical studies and can protect against NAFLD (Tsuda *et al.*, 2003; Jayaprakasam *et al.*, 2006; Guo *et al.*, 2008; Titta *et al.*, 2010; Salamone *et al.*, 2012; Fig. 2). Most studies reporting health benefits of anthocyanins have ignored the impact that they might have on the composition of the microbiota of the GI tract. However, Hidalgo *et al.* (2012) showed relatively strong influences of malvidin 3-glucoside on the microbial composition of pH-controlled faecal (human) batch cultures; the anthocyanin enhanced the growth of all bacteria, but promoted significant increases in *Bifidobacterium* spp. and *Lactobacillus* spp., effectively greater than fructooligosaccharides, which are well-recognized prebiotics. Both Bifids and Lactobacilli are beneficial, reducing the formation of pro-carcinogens, reducing the pH, and promoting the formation of butyrate (an SCFA which has anti-inflammatory and anti-neoplastic effects in the colon; Hidalgo *et al.*, 2012). Similarly, in tests of dietary anthocyanins in berries that limit weight gain of mice on a high-fat diet, antibiotic treatment to remove the microbiota eliminated the preventive effects, suggesting strongly that the impact of anthocyanins on the composition of the gut microbiota represents the primary mechanism whereby they limit weight gain (Esposito *et al.*, 2015). Indeed, berries containing delphinidin-based anthocyanins appear to have stronger effects on weight gain than berries containing cyanidin-based anthocyanins, and cause a substantial shift in GI bacteria away from Firmicutes and towards obligate anaerobes such as Actinobacteria that correlate with decreases in the GI luminal oxygen and oxidative stress (Overall *et al.*, 2017). Clearly, investigation of the impact of anthocyanins on the composition of the microbiota of the GI tract is important to understanding how they promote human health and consequently for dietary recommendations. Moreover, study of their effects on the composition of the gut microbiota in the context of whole foods, and their impact on nutrient/calorie load in humans is important for recommending or designing healthy foods.

Anthocyanins represent just one group of polyphenols with reported effects in combatting obesity and adipogenesis. Other dietary polyphenols have been reported to have beneficial effects on health, including cardio-protection, anti-cancer/pro-apoptosis, anti-inflammatory and neuroprotective effects (Martin *et al.*, 2013; Martin and Li, 2017). Resveratrol, a stilbenoid compound, found in wine (particularly red wine), grape juice, peanuts and blueberries, has effects similar to caloric restriction when consumed as part of the diet, ultimately resulting in weight reduction through prevention of abdominal obesity (Baur *et al.*, 2006; Naderali, 2009; Kim *et al.*, 2011). Dietary resveratrol decreases adipocyte size and increases apoptosis in cell assays (Lekli *et al.*, 2008). However, despite the extensive research on the cellular activities of resveratrol, study of its impact on the microbiota of the GI tract has been very limited. Resveratrol has been shown to increase levels of Lactobacilli and Bifids in rats, implying that some of its beneficial effects on weight gain may operate through modulating the gut microbiota (Larrosa *et al.*, 2009) and it also has antimicrobial activity (Selma *et al.*, 2009). Resveratrol, anthocyanins and flavonols have been found to affect the composition of the microbiota of healthy mice, with the greatest impact when all three polyphenols were combined in a single dietary supplement (Scarano *et al.*, 2017). Compositional changes in the microbiota were associated with reduced symptoms of ulcerative colitis when mice were treated with dextran sodium sulphate (DSS) in a chronic model of gut inflammation (Scarano *et al.*, 2017).

Most fatty acids are associated with positive health outcomes except synthetic trans fats which are strongly associated with sudden cardiac arrest (Table 2).

**Table 2.** The different types of fatty acids in the diet; most are associated with positive health outcomes except synthetic trans fats which are strongly associated with sudden cardiac arrest

Type of fatty acid	Dietary sources	Associated effects on health
Trans fats	Hydrogenated vegetable oils, baked goods, shortening	Strong association with sudden cardiac arrest
Saturated fats	Palm oil, coconut oil, grain-fed animal meats	Historically listed as bad but no clear epidemiological evidence linking consumption to CVD
Monounsaturated fats	Olives, olive oil, canola oil, avocado, nuts, dairy products	High in Mediterranean diet which is beneficial for cardiovascular health
Medium chain omega-6 PUFAs	Safflower, sunflower, corn, cottonseed and soyabean oils	Used in the body to make pro-inflammatory eicosanoids, leading to enhanced inflammation
Medium chain omega-3 PUFAs	Flaxseed, canola, walnut oils, green leafy vegetables	Used in body to make anti-inflammatory eicosanoids and associated with reduced inflammation
Long chain omega-6 PUFAs	Eggs, poultry, pork, liver, farmed fish	Used in the body to make pro-inflammatory eicosanoids, leading to enhanced inflammation
Long chain omega-3 PUFAs	Marine fish including salmon, tuna, sardines, mackerel, herring, shell-fish, pasture-fed cattle and wild game	Used in body to make anti-inflammatory eicosanoids and associated with reduced inflammation

CVD, cardiovascular disease; PUFA, polyunsaturated fatty acid.

## DIFFERENCES IN ABSORPTION AND BIOAVAILABILITY OF PLANT-DERIVED NUTRIENTS

We tend to think of nutrients as single compounds with rather uniform properties, within a group. Even for macronutrients this is a gross oversimplification. Fats, for example, are not uniform in their physical or chemical properties and there are groups of dietary fats with very different (bio)chemical properties and different impacts on health (Table 2).

Polysaccharides include starch, which is composed of α 1–4 linked glucans (amylose and amylopectin) that can be digested at different rates. Fibres are complex, consisting of cellulose (β 1–4 linked glucan), which cannot be digested by humans, and soluble fibres which may be fermented by the microbiota and which may have prebiotic effects through influencing the composition of the microbiota. Generally, bacteria that can ferment oligosaccharides only (*Bifidobacterium* spp. and *Lactobacillus* spp.) offer health-promoting features to the microbiota, and are favoured by oligosaccharide-soluble fibre-rich diets.

Amongst the phytonutrients, there are widely different chemical types within broad chemical groups, with widely different properties which may impact their biological functionalities. For example, more than 600 different anthocyanins have been identified from plants. There is a common assumption that in a dietary context they all behave in the same way (Del Rio *et al.*, 2013), although recent evidence of health-protecting properties being more closely associated with some anthocyanins than others has been offered (Overall *et al.*, 2017). One of the problems associated with studying the health benefits of dietary anthocyanins is their reportedly low bioavailability. Typically, <1 % and often <0.1 % of intact anthocyanins have been reported in plasma in pharmacokinetic studies in both human subjects and animal models. The concentrations detected in peripheral blood are typically in the low nanomolar range, and these are not consistent with the concentrations required to induce biological responses in pre-clinical studies (typically 1–100 μm range). This has led to the notion that the observed effects of consuming anthocyanins are likely to be mediated by anthocyanin metabolites rather than the intact anthocyanins, themselves (Kay, 2006; Kay et al., 2009). Indeed, in human studies by Czank *et al.* (2013) and de Ferrars *et al.* (2014), substantial quantities of ingested labelled anthocyanin ([5-^13^C]-cyanidin-3-glucoside) reached the peripheral circulation in the form of phenolic acid metabolites derived mainly from the anthocyanin B-ring. The total plasma excursion for these phenolic metabolites was 140-fold greater than that for total anthocyanins, with mean concentrations of 3.3 μm compared to 0.14 μm for anthocyanins. More than 20 individual phenolic acid metabolites were identified, including methylated and sulphated metabolites of protocatechuic acid as the major forms. These data support the notion that it is the phenolic acid-derived metabolites of anthocyanins that are likely to induce the changes in cells and tissues that ultimately give rise to beneficial effects in humans, such as improvements in plasma lipid/lipoprotein profiles.

Closely related to the anthocyanins are the flavan 3-ols, which include monomeric forms (catechins and epicatechins) and polymerized forms known as proanthocyanidins. These are formed in plants by reduction of anthocyanidins (to form epicatechins) or leucoanthocyanidins (to form catechins) (Winkle-Shirley, 2001). Epidemiological, clinical and experimental studies have established an inverse correlation between green tea/epicatechin consumption and CVD (Bose *et al.*, 2008). Epicatechins are the major polyphenolic compounds in green tea, and the most significant active component is thought to be epigallocatechin gallate (EGCG). Proanthocyanidins are polymers of catechin and epicatechin and have been reported to be the principal vasoactive polyphenols in red wine (Corder *et al.*, 2001). There are many reports in the literature claiming that proanthocyanins, such as those from red wine and grape seed, offer protection from CVD (Corder *et al.*, 2006; Caton *et al.*, 2010; Gresele *et al.*, 2011). Interestingly, epigallocatechin and delphinidin (anthocyanidin) are structurally very similar, but their *in vivo* metabolites are quite different (Blount *et al.*, 2015). Given that they are reported to have rather similar effects in a dietary context, particularly on protection from CVD, the lack of similarity in their microbial metabolites suggests that these may not be the bioactive forms. Alternatively, their primary effects may be through their impact on the composition of the microbiota, and their metabolites may have little relevance to their bioactivity.

Another example of different vitamers having different bioavailabilities involves vitamin E. Vitamin E activity is conferred by several different tocopherols and tocotrienols made in plants. Bioactivity of dietary vitamin E is influenced heavily by absorption, which is determined by affinity for a transporter in the liver, which is greatest for α-tocopherol. Consequently, the ‘vitamin E’ content of foods is often expressed as ‘α-tocopherol equivalents’, to take into account differences in the uptake and consequently the bioavailability of tocochromanols in plant-based foods (Martin and Li, 2017; Herrera and Barbas, 2001).

As with the examples of dietary polyphenols, differences in bioavailability and/or bioactivity may exist within major groups of other phytonutrients. This is an area that needs far greater definition, but in the absence of recommended daily allowances for many phytonutrients, and without universal acceptance of their roles in protecting health, understanding the bioavailability and bioactivity of individual phytonutrients in this type of detail may be a long way off.

## DIFFERENCES IN THE RESPONSES OF INDIVIDUALS TO PHYTONUTRIENTS

There may be considerable differences between individuals in their responses to plant nutrients, and such differences may confound studies to establish the roles of phytonutrients in protecting the health of consumers. The example of differences between equol producers and non-equol producers has already been described to explain contradictory findings on the health benefits of dietary isoflavones.

In a nutrigenomic observational study of 435 Italian volunteers, of whom 25 % were vegetarian, lipid profiles in plasma were reported to be better for individuals with specific genotypes consuming high polyphenol diets. Rizzi *et al.* (2016) reported significant associations between specific *PON1* alleles, anthocyanin consumption and elevated HDL levels. The *PON1* gene is associated with several human diseases, including CVD (Kim *et al.*, 2013), and is inversely associated with the risk of CVD, particularly with atherosclerosis (Rosenblat *et al.*, 2005). The PON1 enzyme is tightly associated with HDL particles and protects both LDL and HDL from oxidation, a major step in the progression of atherosclerosis (Brophy *et al.*, 2001; Rosenblat *et al.*, 2005; Sahebkar *et al.*, 2016). HDL is a key player in reverse cholesterol transport, which shuttles cholesterol from peripheral cells (e.g. macrophages) to the liver or other tissues. Elevated HDL levels offer a marker for protection against atherosclerosis and CVD. Reverse cholesterol transport that clears cholesterol from the arteries has been reported to respond significantly to dietary anthocyanins in both preclinical and clinical studies (Zhu *et al.*, 2014). While these studies suggest strongly that not all individuals respond in the same way to high polyphenol diets, *PON1* variants could represent useful biomarkers to stratify individuals who might benefit most from targeted dietary recommendations to promote their health.

Recognition that diets may impact differentially the health of individuals has given rise to promotion of the concept of personalized nutrition. In its purest form this would involve the alignment of genomic and lifestyle information for an individual with a diet optimized for their risk factors defined genetically and environmentally. While many see this as an excellent opportunity to make money while tackling the challenges of chronic disease and declining diets, there are considerable logistical barriers towards full implementation, including concerns about data protection, the ‘eating context’ and social acceptance. Indeed, the popularity of the term ‘personalized nutrition’ and its presentation as ‘the way forward’ for dietary improvement has been accompanied by a lack of clarity about what personalized nutrition actually involves (Gibney and Walsh, 2013; Stewart-Knox *et al.*, 2016). This is reflected in the outcomes of the Food4Me project (Celis-Morales *et al.*, 2015), which suggested that providing personalized dietary advice could lead to greater improvements in eating patterns and health outcomes compared to conventional population-based advice. However, the outputs of this survey of personalized advice on nutrition, which claimed to show a genuine impact of personalized nutrition advice on eating patterns, were assessed after only 6 months and showed no added value (in terms of individual adherence to improved diets) between groups that were given generic dietary advice but in a personalized manner and groups that were given personalized dietary advice linked to their phenotype or to their phenotype plus genotype (Livingstone *et al.*, 2016; Table 3). The conclusion of the Food4Me study was that the clinical relevance of this approach was likely to be modest, suggesting that personalized nutrition alone is unlikely to solve the global problems of unhealthy eating, obesity and chronic disease.

**Table 3.** Effect of personalized nutritional advice on adherence to the Mediterranean diet (MedDiet). Redrawn from Livingstone et al., (2016).

		Personalized nutritional advice				Probability		
	Control L0	Personalized nutritional advice (L1+L2+L3)	L1	L2	L3	L0 vs (L1+L2+L3)	L1 vs (L2+L3)	L2 vs L3
Participants (Baseline)	360	1120	373	376	371			
MedDiet Score at Baseline	5.17 ± 0.09	5.10 ± 0.05	5.16 ± 0.09	5.05 ± 0.09	5.09 ± 0.09	0.49	0.36	0.75
MedDiet Score after 6 months	5.20 ± 0.05	5.48 ± 0.07	5.43 ± 0.10	5.38 ± 0.10	5.63 ± 0.10	0.002	0.46	0.029

L0 + Non-personalized dietary advice based on national dietary recommendations, L1 = Personalized advice on basis of current diet and physical activity, L2 = Personalised advice on basis of current diet, physical activity and phenotypic data, L3 = Personalised advice on basis of current diet, physical activity, phenotypic and genotypic data.

Adherence to MedDiet was assessed on the basis of 14-point PREDIMED criteria (Livingstone et al., 2016) relating to (1) A higher intake of olive oil than other cooking oils; (2) A higher intake of white meat compared to other meats; (3) A high intake of fruit, vegetables, olive oil, legumes, nuts, fish, wine and tomato-based sauces; (4) A limited intake of red and processed meats, fats and spreads, sodas, and commercial bakery goods. Participants scored 1 if they met the 14 criteria and 0 if they did not.

## PHYTONUTRIENTS MAY HAVE MULTIPLE MECHANISMS TO PROTECT HEALTH

In addition to the manifold complications associated with establishing the levels of phytonutrients in the foods we eat, how digestible they are, how bioavailable and how bioactive they are, and the differing responses of individuals to consumption of phytonutrients, it is highly unlikely that dietary phytonutrients work by a single mechanism. Many phytonutrients are antioxidants and their antioxidant activity was championed as key to their health-promoting and health-protecting effects by Gutteridge and Halliwell (1994). However, their ability to act as direct antioxidants in humans has been broadly dismissed on the grounds that many phytonutrients have low bioavailability and the efficacy of antioxidant supplements in promoting health has been questioned in systematic reviews (Bjelakovic *et al.*, 2012). Of course, antioxidant activity may be maintained through the microbial metabolites of phytonutrients, and provided these metabolites are bioavailable, the bioactivity of the phytonutrients may be manifest primarily through the antioxidant activity of their metabolites. When considering chronic diseases of the gut, bioavailability is an issue of lesser importance, and it is possible that the anti-inflammatory effects of many dietary polyphenols in the GI tract are the direct result of their antioxidant activities (Tomlinson *et al.*, 2017). Recent results also suggest that the impact of phytonutrients on the composition and metabolic activity of the microbiota is the primary mechanism underpinning their bioactivity. Effects on the gut microbiota could involve the antioxidant capacity of phytonutrients (as many gut microbes are obligate anaerobes) or more diverged activities as identified for different dietary fibres.

A common feature of ‘health-protecting’ phytonutrients is that they have anti-inflammatory activity. The physiological process underpinning many chronic diseases, including CVDs, metabolic diseases and even obesity itself, is believed to be chronic inflammation (Tabas and Glass, 2013; Kaulmann and Bohn, 2014; Pawelec *et al.*, 2014). Chronic inflammation is a complex disorder and although identification of many pharmaceuticals has focused on single key targets in inflammation, the one-target-one-disease approach has matured to view compounds that interfere with multiple targets as superior, in terms of outcomes (Koeberle and Werz, 2014). Clearly, in assessing foods rather than drugs, impacts on multiple inflammation targets are not only possible, but predicted. Therefore, assessing the effects of the phytonutrients broadly on inflammation has become imperative. Such studies would not only give a more generic overview of how different plant-based foods impact health but would also point the way to investigations of how particular plant-based foods offer protection against specific chronic diseases.

## WHY CAN’T WE TAKE SUPPLEMENTS TO ACHIEVE NUTRITIONAL SECURITY?

Many people in developed countries already take dietary supplements to improve their intake of vitamins, micronutrients and omega-3 PUFAs. More than 50 % of Americans use dietary supplements (Bailey *et al.*, 2013) and have been estimated to have spent more than $32 billion on dietary supplements in 2014 (Cohen, 2014). Yet, systematic reviews of the efficacy of supplements suggest that associations between supplement consumption and health outcomes are, at best, inconsistent. A Cochrane review in 2012 of 78 randomized trials with 296 707 participants concluded that antioxidant supplements have little or no effect on health outcomes and an increased risk of mortality associated with consumption of beta-carotene and possibly vitamin E and vitamin A supplements, although none was associated with the use of vitamin C or selenium supplements (Bjelakovic *et al.*, 2012). The Iowa Women’s Health study assessed mortality against supplement use in 38 772 older women (average age 61.6 years) at three time points (1986 baseline; 1997 and 2004) and concluded that several vitamin and mineral supplements were actually associated with increased total mortality risk, with the strongest association for supplemental iron (Mursu *et al.*, 2011; Table 4). Although such studies cannot establish a causal relationship between supplement use and increased mortality, epidemiological studies have failed to provide evidence for benefits of taking multivitamin supplements (Watkins *et al.*, 2000; Neuhouser *et al.*, 2009; Pocobelli *et al.*, 2009; Park *et al.*, 2011). Epidemiological studies of consumption of omega-3 PUFAs have shown positive effects of both dietary and non-dietary (supplement) intake on the incidence of coronary heart disease. However, although there were no statistically significant differences between dietary and supplemental sources of PUFAs, their consumption in diets (fish or seafood) always gave rise to lower risk ratios than consumption of supplements (Bucher *et al.*, 2002). Other studies have shown dietary supplements of omega-3 PUFAs to be effective in high-risk patients only, with no effects in preventing CVD in healthy participants (Marik and Varon, 2009). These authors concluded that supplements should be considered in the secondary prevention of CVD.

**Table 4.** Multivariable adjusted hazard ratio (HR) with 95 % confidence limits (CI) for the dose of iron supplements and the risk of total mortality in women aged 55–69 years at baseline from the IOWA Women’s Health Study

Dose of iron supplement (mg d^–1^)	Cases	HR xx	Cases	HR (95 % CI)	Cases	HR (95 % CI)	Cases	HR (95 % CI)	Cases	HR (95 % CI)
	Non-users		> 0–50		> 50–200		> 200–400		> 400	
Follow-up period										
1986–2008	13 801	1	527	1.02 (0.93–1.12)	222	1.08 (0.94–1.24)	118	1.35 (1.12–1.63)	47	1.57 (1.17–2.11)
1986–1996	3675	1	144	1.09 (0.92–1.30)	59	1.12 (0.86–1.46)	37	1.41 (1.01–1.96)	16	1.70 (1.02–2.83)
1997–2003	2943	1	115	1.13 (0.92–1.39)	74	1.69 (1.33–2.14)	59	1.30 (0.97–1.74)	14	1.91 (1.06–3.45)
2004–2008	1913	1	71	1.66 (1.28–2.14)	71	1.85 (1.43–2.39)	58	1.67 (1.25–2.22)	17	2.01 (1.19–3.40)

The US Preventive Services Task Force (USPSTF) concluded in 2014 that the current evidence is insufficient to assess the balance of benefits and harms of multivitamins or single- or paired-nutrient supplements for the prevention of CVD or cancer. However, the USPSTF recommended against β-carotene or vitamin E supplements for the prevention of CVD or cancer (Moyer, 2014).

In addition to large-scale, epidemiological studies failing to provide clear evidence for the benefits of supplements in protection from chronic disease, some specialized supplements have been linked directly to health problems. One example is OxyElite Pro, a non-prescription supplement which was linked to 97 visits to doctors, 47 hospitalizations, three liver transplants and one death over a 4-month period in Hawaii (Cohen, 2014). Under the Dietary Supplement Health and Education Act (https://www.congress.gov/bill/103rd-congress/senate-bill/784/text) in the USA (1994) labelling of products as a dietary supplement allows it to be sold commercially on the assumption of safety, until proved otherwise. More than 500 supplements in the USA have been found to contain pharmaceutical stimulants and their analogues, but have been sold without studies in humans, as ‘natural products’. Such products, often with inaccurate labelling, underlie many cases of unintentional ‘doping’ and failed drugs tests at sporting events. A 2015 analysis suggested that 23 000 emergency department visits to hospitals and health centres in the USA could be attributed to adverse effects, particularly cardiovascular events, resulting from consumption of dietary supplements (Geller *et al.*, 2015).

Furthermore, many dietary supplements do not contain the nutrients they claim. For example, the content of ephedra alkaloids in herbal dietary supplements containing ephedra was assayed in one study (Gurley *et al.*, 2000). Assays of 20 ephedra-containing dietary supplements showed that alkaloid content differed substantially from label claims in more than 50 % of the products and in some cases were inconsistent between two batches of the same product. One product contained no ephedra alkaloids at all, whereas others had contents varying by more than 20 % of those indicated on the label.

Finally, for some phytonutrients, at least, there is evidence that they do not have the same beneficial effects when consumed in extracted/purified forms compared to their activities in whole foods (Eberhardt *et al.*, 2000; Liu, 2003, 2004, 2013; de Kok *et al.*, 2008; Prior *et al.*, 2008; Titta *et al.*, 2010). This is probably because the food matrix is an important component in bioactivity, influencing absorption, microbial metabolism, synergistic interactions between different phytonutrients and effects on the composition of the GI microbiota. For all these reasons, improving health outcomes and protection against chronic diseases needs to be based on dietary improvements and increased consumption of plant-based foods, particularly fresh fruit and vegetables, rather than improvement of low-quality Western diets with supplements that may not work, or may, indeed, increase the risk of disease.

## CONCLUSIONS

**Fig. 3.**
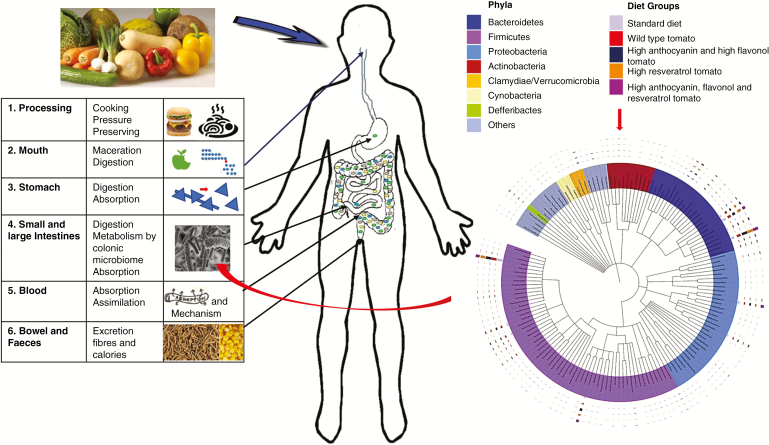
Summary diagram of the processes impacting plants and plant-based foods in the diet. Processing of foods impacts their chemistry, physical properties and digestibility. Once ingested, maceration and digestion by amylases, proteases, lipases, etc., impact the bioavailability of macronutrients, micronutrients and phytonutrients. Dietary nutrients may impact the composition and functioning of the GI microbiota, which predominantly resides in the colon. Changing microbiota functionality may impact the further digestion of food, nutrient absorption, nutrient metabolism in the colon, removal of toxins and pathogens and signalling, especially satiation. Nutrients, or their metabolites, will be absorbed through the gut and enter the bloodstream, where they may have a wide range of physiological effects. Some material, particularly insoluble fibre, will be excreted. Undigested nutrients will also be excreted and may limit the calories and nutrients redeemable from foods. The panel on the right shows the complexity of the different bacterial phyla in the microbiota of mice using a circular phylogenetic tree colour-coded for the different phyla. The bars above the genera summarize the impact of diets enriched in regular tomatoes (red), high anthocyanin and flavonol tomatoes (dark blue), high resveratrol tomatoes (orange) and high anthocyanin, flavonol and resveratrol tomatoes (purple) compared to the standard diet (grey) on the composition of the microbiota. A large bar of the colour of the supplemented diets above each bacterial group that was particularly enriched by specific polyphenol-rich diets is shown (from Scarano *et al.*, 2017).

What I have tried to do in this article is to explain, by example, some of the complexities that impact our understanding of the beneficial role of plant-based foods in our diets. These are summarized in Fig. 3. The six different processes impacting food digestion and nutrient assimilation shown in Fig. 3 require substantial investment in research if we are to understand the relationship between plants, our diet and our health. The outcome of greater understanding, particularly of the mechanisms and relative efficacies of different phytonutrients on protection from chronic disease, would go a long way towards persuading the agricultural and food sectors of the importance of nutritional security, and to set aside current emphases on yield, profit margins and processed cheap foods. Such changes would benefit societies, the world over. If this does not happen, and the agricultural and food sectors continue to lobby against measures to improve nutrition (http://time.com/4130043/lobbying-politics-dietary-guidelines;https://www.foodmanufacture.co.uk/Article/2016/06/20/UK-Obesity-plans-stalled-by-food-and-drink-industry;Nestle, 2013), especially that of children, perhaps consumers will turn to a new generation of growers, producers and food manufacturers that do prioritize the nutritional quality of their products. Certainly, it would be expedient for politicians and policy-makers, already struggling with rising healthcare costs associated with the increasing numbers of people living with chronic disease, to support research by plant scientists, through multidisciplinary collaborations, into the roles of plant-based foods in health and well-being, and thereby to support progress towards ensuring global nutritional security.
